# 2,4-Diamino-5-(4-chloro­phen­yl)-6-ethyl­pyrimidin-1-ium 2-acet­amido­benzoate

**DOI:** 10.1107/S1600536811032570

**Published:** 2011-08-17

**Authors:** Sampath Natarajan, Rita Mathews

**Affiliations:** a Department of Advanced Technology Fusion, Konkuk University, 1 Hwayang-dong, Gwangjin-gu, Seoul 143 701, Republic of Korea

## Abstract

The title compound, C_12_H_14_ClN_4_^+^·C_9_H_8_NO_3_^−^, is a salt with a 1:1 ratio of cation and anion components inter­acting with each other forming an *R*_2_^2^(8) ring motif. The crystal structure is stabilized by hydrogen bonds (N—H⋯O) involving two different eight-membered rings. One of them is formed between the pyrimidine ring (donor) and the carboxylate group (acceptor) from the benzoate, whereas the other ring is formed by N—H⋯O interactions, which help to form a dimer between two symmetry-related salts in the unit cell. In addition, an intramolecular C—H⋯N and intermolecular C—H⋯Cl interactions help to control the molecules in the unit-cell packing.

## Related literature

For related literature on amino­pyrimidine–carboxyl­ate inter­actions, see: Baker & Santi (1965 ▶); Chinnakali *et al.* (1999 ▶); Desiraju (1989 ▶); Hunt *et al.* (1980 ▶); Lynch & Jones (2004 ▶); Stanley *et al.* (2005 ▶). For literature on amino­pyrimidine and benzoic acid adducts, see: Thanigaimani *et al.* (2006 ▶, 2007 ▶); Balasubramani *et al.* (2005 ▶, 2006 ▶). For puckering parameters, see: Cremer & Pople, (1975 ▶); Nardelli (1995 ▶).
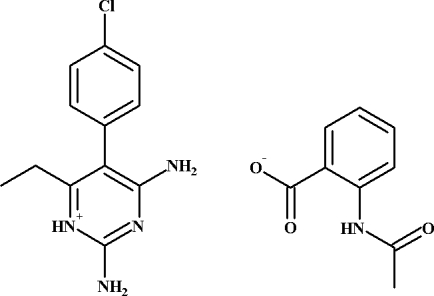


## Experimental

### 

#### Crystal data

C_12_H_14_ClN_4_^+^·C_9_H_8_NO_3_^−^
*M* *_r_* = 427.89
Monoclinic, 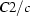

*a* = 25.226 (4) Å
*b* = 9.0666 (16) Å
*c* = 20.688 (4) Å
β = 113.400 (3)°
*V* = 4342.5 (13) Å^3^
*Z* = 8
Mo *K*α radiation
μ = 0.21 mm^−1^
*T* = 293 K
0.4 × 0.35 × 0.32 mm

#### Data collection

Bruker SMART APEX CCD area-detector diffractometer
14400 measured reflections
3937 independent reflections
3079 reflections with *I* > 2σ(*I*)
*R*_int_ = 0.019

#### Refinement

*R*[*F* ^2^ > 2σ(*F* ^2^)] = 0.046
*wR*(*F* ^2^) = 0.123
*S* = 1.04
3937 reflections
272 parameters
H-atom parameters constrained
Δρ_max_ = 0.20 e Å^−3^
Δρ_min_ = −0.19 e Å^−3^

### 

Data collection: *SMART* (Bruker, 2004 ▶); cell refinement: *SAINT* (Bruker, 2004 ▶); data reduction: *SAINT*; program(s) used to solve structure: *SHELXS97* (Sheldrick, 2008 ▶); program(s) used to refine structure: *SHELXL97* (Sheldrick, 2008 ▶); molecular graphics: *ORTEP-3* (Farrugia, 1997 ▶); software used to prepare material for publication: *PLATON* (Spek, 2009 ▶).

## Supplementary Material

Crystal structure: contains datablock(s) I, global. DOI: 10.1107/S1600536811032570/ng5209sup1.cif

Structure factors: contains datablock(s) I. DOI: 10.1107/S1600536811032570/ng5209Isup2.hkl

Supplementary material file. DOI: 10.1107/S1600536811032570/ng5209Isup3.cml

Additional supplementary materials:  crystallographic information; 3D view; checkCIF report

## Figures and Tables

**Table 1:** Hydrogen-bond geometry (Å, °)

*D*—H⋯*A*	*D*—H	H⋯*A*	*D*⋯*A*	*D*—H⋯*A*
N5—H5⋯O1	0.86	1.90	2.614 (2)	140
C9—H9*B*⋯N5	0.97	2.87	3.683 (3)	142
N1—H1⋯O1^i^	0.86	1.86	2.715 (2)	177
N3—H3*B*⋯O2^i^	0.86	1.96	2.802 (3)	165
N3—H3*A*⋯O2^ii^	0.86	2.16	2.897 (2)	143
N4—H4*A*⋯O3^iii^	0.86	2.01	2.859 (2)	168
C10—H10*B*⋯Cl1^iv^	0.96	2.95	3.864 (3)	160

Symmetry codes: (i) 
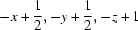
; (ii) 
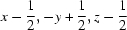
; (iii) 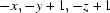; (iv) 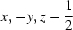.

**Fig. 1.**
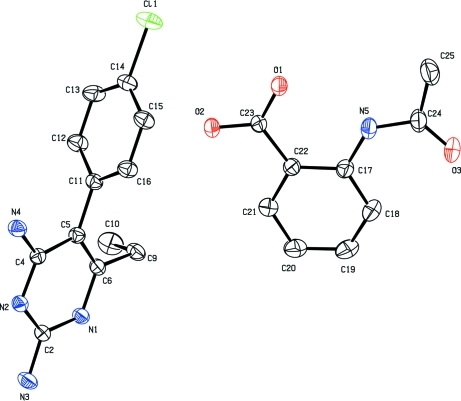
ORTEP diagram of the title molecule with the atom numbering scheme. Displacement ellipsoid are drawn at 30% probability level.

**Fig. 2.**
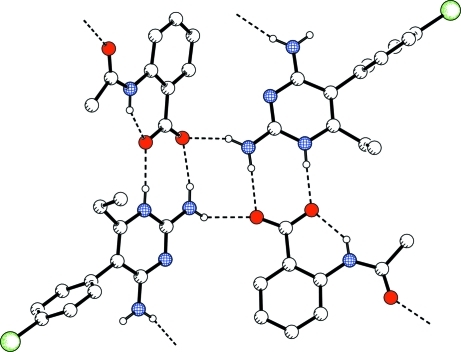
Dimer interaction between the symmetry related salts of title compound. Dashed lines indicate the intra and intermolecular hydrogen bonds.

**Fig. 3.**
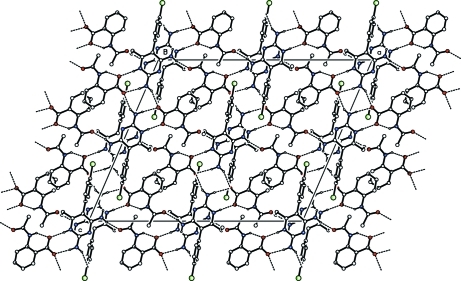
Packing diagram of the title compound viewed down the b axis. Dashed lines indicate the intra and intermolecular interactions between the molecules.

## supplementary crystallographic information

### Comment

Aminopyrimidine-Carboxylate interactions are important since they are involved in protein-nucleic acids recognition and protein-drug binding. Hydrogen bonding plays a key role in molecular recognition and crystal engineering research (Desiraju, 1989). In general, aminopyrimidines posses self complementary hydrogen-bonded motifs forming a base pair which in itself is a unique property. The adducts of carboxylic acid with 2-aminopyrimidine system form a graph-set motif *R*_2_^2^(8) (Lynch & Jones, 2004). This motif is very robust in aminopyrimidine-carboxylic acid/carboxylates systems. The crystal structures of many aminopyrimidine carboxylates (Stanley *et al.*, 2005) and co-crystal structures (Chinnakali *et al.*,1999) have been reported. Many structures of aminopyrimidine and benzoic acid adducts have been reported. Few of them are 2-amino-4,6-dimethoxy pyrimidine: 4-aminobenzoic acid (Thanigaimani *et al.*, 2006), 2-amino-4,6-dimethoxypyrimidine: phthalic acid (Thanigaimani *et al.*, 2007), 2-amino-4,6-dimethylpyrimidine: cinnamic acid (Balasubramani *et al.*, 2005) and 2-amino-4,6-dimethylpyrimidine: 4-hydroxybenzoic acid (Balasubramani *et al.*, 2006). All these reported structures also have common features of heterosynthone formation. In the present study we report a salt (1:1) namely, 2,4-diamino-5-(4-chlorophenyl)-6-ethylpyrimidin-1-ium- 2-(acetylamino)benzoate and its interactions are studied extensively.

The asymmetric unit of crystal contains a single molecule of each component of salt (Fig. 1) and does not have any direct interactions each other. The interactions were found between the symmetry related molecules of aminopyrimidin-1-ium and benzoate salt *via* hydrogen bonds N1—H1···O1 and N3—H3B···O2 (-*x* + 1/2, -*y* + 1/2, -*z* + 1) (Fig. 2). Here the pyrimidine acts as a donor which donates two H atoms and carboxylate O atoms as acceptors. In addition dimeric interactions by the atoms O2 and N3 form an eight membered ring through the Hydrogen bonds N3—H3A···O2 (*x* - 1/2, -*y* + 1/2, *z* - 1/2) and N3—H3B···O2 (-*x* + 1/2, -*y* + 1/2, -*z* + 1) (Fig. 2). The dihedral angle between the rings, 4-chlorophenyl and 2,4-diaminopyrimidine is 80.6 (1)°. This value is higher than that in a biphenyl ring system. This may be due to the substitution of ethyl and amine groups at C4 and C6, respectively. An extended moiety of acetylamino group is coplanar with phenyl carboxylate and the dihedral angle between these two is 1.59 (1)° (Cremer & Pople, 1975; Nardelli, 1995).

In the crystal packing, the molecules are arranged as sheets along the a direction (Fig. 3). These sheets are organized as two layers in the unit cell and both layer molecules are connected to each other through N4—H4A···O3 hydrogen bond. In addition, a six membered ring is formed by an intra-molecular interaction (N5—H5···O1)in benzoate molecule which also controls the molecules in crystal packing. Molecular packing is stabilized by many N—H···O and a C—H···Cl (*x*, -*y*, *z* - 1/2) intra and intermolecular interactions (Table 1, Fig. 2).

### Experimental

A hot methanolic solution (20 ml) of 2,4-diamino-5-(4-chlorophenyl)-6- ethylpyrimidine and 2-(acetylamino)benzoic acid in the ratio of 1:1 was warmed for 0.5 h over a water bath. The mixture was cooled slowly and kept at room temperature and after a few days, colourless crystals were obtained

### Refinement

H atoms were positioned geometrically and refined using a riding model with C—H = 0.93 Å for aromatic H, 0.97 Å for methylene, 0.96 Å for methyl H atoms and for aromatic NH_2_ and N—H = 0.86 Å. The *U*_iso_ parameters for H atoms were constraned to be 1.5Ueq of the carrier atom for the methyl H atoms and 1.2Ueq of the carrier atom for the remaining H atoms.

### Crystal data

**Table:**

C_12_H_14_ClN_4_^+^·C_9_H_8_NO_3_^−^	*F*(000) = 1792
*M**_r_* = 427.89	*D*_x_ = 1.309 Mg m^−^^3^
Monoclinic, *C*2/*c*	Mo *K*α radiation, λ = 0.71073 Å
*a* = 25.226 (4) Å	Cell parameters from 14400 reflections
*b* = 9.0666 (16) Å	θ = 1.8–26.2°
*c* = 20.688 (4) Å	µ = 0.21 mm^−^^1^
β = 113.400 (3)°	*T* = 293 K
*V* = 4342.5 (13) Å^3^	Block, colourless
*Z* = 8	0.4 × 0.35 × 0.32 mm

### Data collection

**Table:**

Bruker SMART APEX CCD area-detector diffractometer	3079 reflections with *I* > 2σ(*I*)
Radiation source: fine-focus sealed tube	*R*_int_ = 0.019
graphite	θ_max_ = 26.2°, θ_min_ = 1.8°
ω scans	*h* = −29→30
14400 measured reflections	*k* = −11→11
3937 independent reflections	*l* = −25→24

### Refinement

**Table:**

Refinement on *F*^2^	Primary atom site location: structure-invariant direct methods
Least-squares matrix: full	Secondary atom site location: difference Fourier map
*R*[*F*^2^ > 2σ(*F*^2^)] = 0.046	Hydrogen site location: inferred from neighbouring sites
*wR*(*F*^2^) = 0.123	H-atom parameters constrained
*S* = 1.04	*w* = 1/[σ^2^(*F*_o_^2^) + (0.059*P*)^2^ + 2.2497*P*] where *P* = (*F*_o_^2^ + 2*F*_c_^2^)/3
3937 reflections	(Δ/σ)_max_ < 0.001
272 parameters	Δρ_max_ = 0.20 e Å^−^^3^
0 restraints	Δρ_min_ = −0.19 e Å^−^^3^

### Special details

**Table:**

Geometry. All e.s.d.'s (except the e.s.d. in the dihedral angle between two l.s. planes) are estimated using the full covariance matrix. The cell e.s.d.'s are taken into account individually in the estimation of e.s.d.'s in distances, angles and torsion angles; correlations between e.s.d.'s in cell parameters are only used when they are defined by crystal symmetry. An approximate (isotropic) treatment of cell e.s.d.'s is used for estimating e.s.d.'s involving l.s. planes.
Refinement. Refinement of *F*^2^ against ALL reflections. The weighted *R*-factor *wR* and goodness of fit *S* are based on *F*^2^, conventional *R*-factors *R* are based on *F*, with *F* set to zero for negative *F*^2^. The threshold expression of *F*^2^ > σ(*F*^2^) is used only for calculating *R*-factors(gt) *etc*. and is not relevant to the choice of reflections for refinement. *R*-factors based on *F*^2^ are statistically about twice as large as those based on *F*, and *R*- factors based on ALL data will be even larger.

### Fractional atomic coordinates and isotropic or equivalent isotropic displacement parameters (Å^2^)

**Table:**

	*x*	*y*	*z*	*U*_iso_*/*U*_eq_	
Cl1	0.09641 (3)	0.17665 (8)	0.85360 (3)	0.0916 (3)	
O1	0.34974 (6)	0.30249 (17)	0.60241 (7)	0.0668 (4)	
O2	0.40391 (6)	0.20074 (17)	0.70432 (7)	0.0651 (4)	
O3	0.16543 (8)	0.5398 (3)	0.55466 (10)	0.1088 (7)	
N1	0.06713 (6)	0.24269 (18)	0.44774 (7)	0.0487 (4)	
H1	0.0925	0.2276	0.4304	0.058*	
N5	0.25286 (7)	0.4296 (2)	0.58885 (9)	0.0691 (5)	
H5	0.2795	0.4091	0.5742	0.083*	
N2	−0.02695 (6)	0.31925 (17)	0.42880 (8)	0.0490 (4)	
C2	0.01378 (8)	0.2902 (2)	0.40505 (9)	0.0473 (4)	
C4	−0.01287 (7)	0.30452 (19)	0.49808 (9)	0.0449 (4)	
C5	0.04201 (8)	0.2494 (2)	0.54570 (9)	0.0450 (4)	
C6	0.08133 (8)	0.2183 (2)	0.51771 (9)	0.0458 (4)	
N3	0.00325 (7)	0.3063 (2)	0.33758 (8)	0.0642 (5)	
H3A	−0.0302	0.3358	0.3086	0.077*	
H3B	0.0298	0.2873	0.3226	0.077*	
N4	−0.05259 (7)	0.34253 (19)	0.52206 (8)	0.0573 (4)	
H4A	−0.0856	0.3749	0.4934	0.069*	
H4B	−0.0453	0.3347	0.5662	0.069*	
C9	0.13931 (8)	0.1497 (2)	0.55671 (10)	0.0573 (5)	
H9A	0.1505	0.1627	0.6069	0.069*	
H9B	0.1678	0.1987	0.5437	0.069*	
C10	0.13821 (12)	−0.0142 (3)	0.54001 (14)	0.0866 (8)	
H10A	0.1758	−0.0557	0.5655	0.130*	
H10B	0.1277	−0.0270	0.4904	0.130*	
H10C	0.1105	−0.0630	0.5536	0.130*	
C11	0.05491 (8)	0.2300 (2)	0.62204 (9)	0.0461 (4)	
C12	0.05200 (10)	0.0934 (2)	0.65023 (10)	0.0615 (5)	
H12	0.0415	0.0115	0.6208	0.074*	
C13	0.06433 (10)	0.0761 (2)	0.72089 (11)	0.0672 (6)	
H13	0.0625	−0.0165	0.7392	0.081*	
C14	0.07937 (9)	0.1978 (2)	0.76379 (10)	0.0583 (5)	
C15	0.08186 (10)	0.3350 (2)	0.73762 (10)	0.0624 (5)	
H15	0.0916	0.4169	0.7672	0.075*	
C16	0.06970 (9)	0.3503 (2)	0.66685 (10)	0.0565 (5)	
H16	0.0715	0.4433	0.6489	0.068*	
C17	0.26374 (9)	0.3814 (2)	0.65760 (11)	0.0597 (5)	
C18	0.22574 (11)	0.4080 (3)	0.69018 (15)	0.0842 (7)	
H18	0.1915	0.4589	0.6659	0.101*	
C19	0.23812 (13)	0.3602 (4)	0.75731 (16)	0.0925 (8)	
H19	0.2122	0.3790	0.7781	0.111*	
C20	0.28810 (12)	0.2852 (3)	0.79432 (14)	0.0819 (7)	
H20	0.2964	0.2536	0.8401	0.098*	
C21	0.32583 (10)	0.2572 (3)	0.76267 (11)	0.0654 (6)	
H21	0.3597	0.2059	0.7878	0.078*	
C22	0.31507 (8)	0.3029 (2)	0.69465 (10)	0.0516 (5)	
C23	0.35930 (8)	0.2663 (2)	0.66497 (10)	0.0498 (5)	
C24	0.20715 (10)	0.5028 (3)	0.54250 (13)	0.0763 (7)	
C25	0.21049 (12)	0.5345 (4)	0.47279 (14)	0.0992 (9)	
H25A	0.1732	0.5193	0.4355	0.149*	
H25B	0.2381	0.4696	0.4664	0.149*	
H25C	0.2223	0.6350	0.4720	0.149*	

### Atomic displacement parameters (Å^2^)

**Table:**

	*U*^11^	*U*^22^	*U*^33^	*U*^12^	*U*^13^	*U*^23^
Cl1	0.1155 (6)	0.1148 (6)	0.0436 (3)	0.0138 (4)	0.0307 (3)	0.0113 (3)
O1	0.0521 (8)	0.0950 (11)	0.0544 (9)	0.0212 (8)	0.0222 (7)	0.0160 (8)
O2	0.0497 (8)	0.0881 (11)	0.0533 (8)	0.0187 (7)	0.0158 (6)	0.0105 (7)
O3	0.0666 (11)	0.1441 (18)	0.0979 (13)	0.0506 (12)	0.0139 (10)	0.0016 (12)
N1	0.0421 (8)	0.0635 (10)	0.0400 (8)	0.0054 (7)	0.0159 (6)	0.0034 (7)
N5	0.0487 (9)	0.0840 (13)	0.0700 (12)	0.0178 (9)	0.0187 (8)	0.0095 (10)
N2	0.0410 (8)	0.0601 (10)	0.0426 (8)	0.0025 (7)	0.0131 (7)	0.0051 (7)
C2	0.0437 (10)	0.0538 (11)	0.0400 (9)	−0.0003 (8)	0.0118 (8)	0.0027 (8)
C4	0.0432 (10)	0.0455 (10)	0.0449 (10)	−0.0014 (8)	0.0164 (8)	0.0034 (8)
C5	0.0461 (10)	0.0441 (10)	0.0428 (9)	0.0009 (8)	0.0156 (8)	0.0028 (8)
C6	0.0445 (10)	0.0488 (10)	0.0391 (9)	−0.0005 (8)	0.0112 (8)	−0.0002 (8)
N3	0.0499 (9)	0.1003 (14)	0.0388 (9)	0.0151 (9)	0.0136 (7)	0.0090 (8)
N4	0.0454 (9)	0.0785 (12)	0.0478 (9)	0.0105 (8)	0.0183 (7)	0.0101 (8)
C9	0.0496 (11)	0.0754 (14)	0.0418 (10)	0.0112 (10)	0.0128 (8)	0.0048 (9)
C10	0.0947 (18)	0.0754 (16)	0.0860 (17)	0.0288 (14)	0.0320 (14)	0.0133 (13)
C11	0.0449 (10)	0.0496 (11)	0.0420 (9)	0.0060 (8)	0.0154 (8)	0.0035 (8)
C12	0.0830 (15)	0.0490 (12)	0.0509 (11)	−0.0012 (10)	0.0248 (10)	0.0001 (9)
C13	0.0921 (16)	0.0565 (13)	0.0550 (12)	0.0053 (11)	0.0314 (11)	0.0141 (10)
C14	0.0613 (12)	0.0722 (14)	0.0407 (10)	0.0115 (10)	0.0194 (9)	0.0065 (10)
C15	0.0770 (14)	0.0601 (13)	0.0473 (11)	0.0038 (11)	0.0218 (10)	−0.0065 (9)
C16	0.0703 (13)	0.0474 (11)	0.0512 (11)	0.0036 (9)	0.0235 (10)	0.0034 (9)
C17	0.0486 (11)	0.0663 (13)	0.0625 (12)	0.0024 (9)	0.0204 (9)	−0.0077 (10)
C18	0.0624 (14)	0.1020 (19)	0.0919 (19)	0.0213 (13)	0.0344 (13)	−0.0068 (15)
C19	0.0876 (19)	0.121 (2)	0.0872 (19)	0.0119 (17)	0.0543 (16)	−0.0122 (17)
C20	0.0856 (18)	0.104 (2)	0.0663 (14)	0.0015 (15)	0.0405 (13)	−0.0067 (14)
C21	0.0640 (13)	0.0748 (14)	0.0574 (12)	0.0038 (11)	0.0243 (10)	−0.0023 (11)
C22	0.0447 (10)	0.0536 (11)	0.0539 (11)	−0.0036 (8)	0.0169 (8)	−0.0081 (9)
C23	0.0430 (10)	0.0543 (11)	0.0475 (11)	0.0014 (8)	0.0129 (8)	−0.0032 (9)
C24	0.0526 (13)	0.0783 (16)	0.0795 (16)	0.0147 (12)	0.0065 (11)	−0.0024 (12)
C25	0.0818 (17)	0.111 (2)	0.0846 (18)	0.0232 (16)	0.0111 (14)	0.0237 (16)

### Geometric parameters (Å, °)

**Table:**

Cl1—C14	1.7433 (19)	C10—H10C	0.9600
O1—C23	1.262 (2)	C11—C12	1.383 (3)
O2—C23	1.247 (2)	C11—C16	1.383 (3)
O3—C24	1.222 (3)	C12—C13	1.377 (3)
N1—C2	1.354 (2)	C12—H12	0.9300
N1—C6	1.364 (2)	C13—C14	1.372 (3)
N1—H1	0.8600	C13—H13	0.9300
N5—C24	1.346 (3)	C14—C15	1.368 (3)
N5—C17	1.407 (3)	C15—C16	1.379 (3)
N5—H5	0.8600	C15—H15	0.9300
N2—C2	1.329 (2)	C16—H16	0.9300
N2—C4	1.339 (2)	C17—C18	1.395 (3)
C2—N3	1.321 (2)	C17—C22	1.408 (3)
C4—N4	1.328 (2)	C18—C19	1.367 (4)
C4—C5	1.433 (2)	C18—H18	0.9300
C5—C6	1.362 (3)	C19—C20	1.368 (4)
C5—C11	1.491 (2)	C19—H19	0.9300
C6—C9	1.497 (3)	C20—C21	1.376 (3)
N3—H3A	0.8600	C20—H20	0.9300
N3—H3B	0.8600	C21—C22	1.386 (3)
N4—H4A	0.8600	C21—H21	0.9300
N4—H4B	0.8600	C22—C23	1.509 (3)
C9—C10	1.523 (3)	C24—C25	1.505 (4)
C9—H9A	0.9700	C25—H25A	0.9600
C9—H9B	0.9700	C25—H25B	0.9600
C10—H10A	0.9600	C25—H25C	0.9600
C10—H10B	0.9600		
			
C2—N1—C6	121.21 (16)	C14—C13—C12	118.9 (2)
C2—N1—H1	119.4	C14—C13—H13	120.6
C6—N1—H1	119.4	C12—C13—H13	120.6
C24—N5—C17	129.9 (2)	C15—C14—C13	121.32 (18)
C24—N5—H5	115.1	C15—C14—Cl1	119.43 (17)
C17—N5—H5	115.1	C13—C14—Cl1	119.25 (17)
C2—N2—C4	117.42 (14)	C14—C15—C16	119.05 (19)
N3—C2—N2	120.54 (16)	C14—C15—H15	120.5
N3—C2—N1	117.07 (17)	C16—C15—H15	120.5
N2—C2—N1	122.39 (16)	C15—C16—C11	121.32 (19)
N4—C4—N2	117.08 (15)	C15—C16—H16	119.3
N4—C4—C5	120.00 (16)	C11—C16—H16	119.3
N2—C4—C5	122.92 (16)	C18—C17—N5	122.7 (2)
C6—C5—C4	116.60 (16)	C18—C17—C22	119.0 (2)
C6—C5—C11	122.45 (16)	N5—C17—C22	118.38 (18)
C4—C5—C11	120.95 (16)	C19—C18—C17	120.8 (2)
C5—C6—N1	119.27 (16)	C19—C18—H18	119.6
C5—C6—C9	125.29 (16)	C17—C18—H18	119.6
N1—C6—C9	115.35 (16)	C18—C19—C20	120.9 (2)
C2—N3—H3A	120.0	C18—C19—H19	119.5
C2—N3—H3B	120.0	C20—C19—H19	119.5
H3A—N3—H3B	120.0	C19—C20—C21	118.9 (2)
C4—N4—H4A	120.0	C19—C20—H20	120.6
C4—N4—H4B	120.0	C21—C20—H20	120.6
H4A—N4—H4B	120.0	C20—C21—C22	122.3 (2)
C6—C9—C10	110.93 (18)	C20—C21—H21	118.8
C6—C9—H9A	109.5	C22—C21—H21	118.8
C10—C9—H9A	109.5	C21—C22—C17	118.07 (19)
C6—C9—H9B	109.5	C21—C22—C23	117.99 (17)
C10—C9—H9B	109.5	C17—C22—C23	123.93 (18)
H9A—C9—H9B	108.0	O2—C23—O1	123.28 (18)
C9—C10—H10A	109.5	O2—C23—C22	117.42 (17)
C9—C10—H10B	109.5	O1—C23—C22	119.29 (16)
H10A—C10—H10B	109.5	O3—C24—N5	123.6 (3)
C9—C10—H10C	109.5	O3—C24—C25	121.7 (2)
H10A—C10—H10C	109.5	N5—C24—C25	114.8 (2)
H10B—C10—H10C	109.5	C24—C25—H25A	109.5
C12—C11—C16	117.94 (17)	C24—C25—H25B	109.5
C12—C11—C5	121.79 (17)	H25A—C25—H25B	109.5
C16—C11—C5	120.26 (17)	C24—C25—H25C	109.5
C13—C12—C11	121.50 (19)	H25A—C25—H25C	109.5
C13—C12—H12	119.3	H25B—C25—H25C	109.5
C11—C12—H12	119.3		
			
C4—N2—C2—N3	−178.21 (17)	C12—C13—C14—Cl1	−178.78 (17)
C4—N2—C2—N1	2.2 (3)	C13—C14—C15—C16	−0.9 (3)
C6—N1—C2—N3	−177.84 (17)	Cl1—C14—C15—C16	178.42 (17)
C6—N1—C2—N2	1.8 (3)	C14—C15—C16—C11	0.2 (3)
C2—N2—C4—N4	175.89 (16)	C12—C11—C16—C15	0.7 (3)
C2—N2—C4—C5	−4.6 (3)	C5—C11—C16—C15	−179.95 (19)
N4—C4—C5—C6	−177.47 (17)	C24—N5—C17—C18	−1.4 (4)
N2—C4—C5—C6	3.1 (3)	C24—N5—C17—C22	178.4 (2)
N4—C4—C5—C11	1.7 (3)	N5—C17—C18—C19	−179.5 (2)
N2—C4—C5—C11	−177.80 (16)	C22—C17—C18—C19	0.7 (4)
C4—C5—C6—N1	1.0 (3)	C17—C18—C19—C20	0.0 (5)
C11—C5—C6—N1	−178.14 (16)	C18—C19—C20—C21	−0.5 (5)
C4—C5—C6—C9	−175.26 (17)	C19—C20—C21—C22	0.2 (4)
C11—C5—C6—C9	5.6 (3)	C20—C21—C22—C17	0.5 (3)
C2—N1—C6—C5	−3.3 (3)	C20—C21—C22—C23	−179.9 (2)
C2—N1—C6—C9	173.28 (17)	C18—C17—C22—C21	−0.9 (3)
C5—C6—C9—C10	100.7 (2)	N5—C17—C22—C21	179.27 (19)
N1—C6—C9—C10	−75.7 (2)	C18—C17—C22—C23	179.5 (2)
C6—C5—C11—C12	−80.4 (2)	N5—C17—C22—C23	−0.3 (3)
C4—C5—C11—C12	100.5 (2)	C21—C22—C23—O2	−2.1 (3)
C6—C5—C11—C16	100.3 (2)	C17—C22—C23—O2	177.44 (19)
C4—C5—C11—C16	−78.8 (2)	C21—C22—C23—O1	177.81 (18)
C16—C11—C12—C13	−1.1 (3)	C17—C22—C23—O1	−2.7 (3)
C5—C11—C12—C13	179.58 (19)	C17—N5—C24—O3	−0.3 (4)
C11—C12—C13—C14	0.5 (3)	C17—N5—C24—C25	−179.4 (2)
C12—C13—C14—C15	0.5 (3)		

### Hydrogen-bond geometry (Å, °)

**Table:**

*D*—H···*A*	*D*—H	H···*A*	*D*···*A*	*D*—H···*A*
N5—H5···O1	0.86	1.90	2.614 (2)	140
C9—H9B···N5	0.97	2.87	3.683 (3)	142
N1—H1···O1^i^	0.86	1.86	2.715 (2)	177
N3—H3B···O2^i^	0.86	1.96	2.802 (3)	165
N3—H3A···O2^ii^	0.86	2.16	2.897 (2)	143
N4—H4A···O3^iii^	0.86	2.01	2.859 (2)	168
C10—H10B···Cl1^iv^	0.96	2.95	3.864 (3)	160

Symmetry codes: (i) −*x*+1/2, −*y*+1/2, −*z*+1; (ii) *x*−1/2, −*y*+1/2, *z*−1/2; (iii) −*x*, −*y*+1, −*z*+1; (iv) *x*, −*y*, *z*−1/2.
